# Neurovaskuläre Manifestationen von COVID‑19

**DOI:** 10.1007/s00115-021-01104-1

**Published:** 2021-03-24

**Authors:** Roland Veltkamp, Jan C. Purrucker, Ralph Weber

**Affiliations:** 1 Neurologische Klinik, Alfried Krupp Krankenhaus Essen, Alfried Krupp Str. 21, 45131 Essen, Deutschland; 2 Department of Brain Sciences, Imperial College London, London, Großbritannien; 3 Neurologische Klinik, Universitätsklinikum Heidelberg, Heidelberg, Deutschland

**Keywords:** SARS-CoV‑2, Zerebrale Ischämie, Intrazerebrale Blutung, Thrombolyse, Thrombektomie, SARS-CoV‑2, Cerebral ischemia, Intracerebral hemorrhage, Thrombolysis, Thrombectomy

## Abstract

Bereits früh nach Beginn der COVID‑19(„coronavirus disease 2019“)-Pandemie wurden Schlaganfälle als Manifestation oder Komplikation einer SARS-CoV-2(„severe acute respiratory syndrome coronavirus 2“)-Infektion beschrieben. Aktuelle Metaanalysen berichten eine Schlaganfallrate von etwa 1,5 %. Schlaganfälle bei COVID‑19-Patienten treten zwar häufiger bei schweren Verläufen der Infektion und bei älteren Patienten mit kardiovaskulären Risiken auf. Nicht selten sind aber auch junge Patienten ohne Risikofaktoren betroffen. Die Schlaganfallmechanismen sind vorwiegend embolisch, die Thromben verschließen häufig große intrakranielle Gefäße und betreffen in mehr als 20 % mehrere Gefäßterritorien, während mikroangiopathische Infarkte selten sind. Die genaue Emboliequelle bleibt bei über 40 % der Betroffenen kryptogen. Die durch das Zusammentreffen einer SARS-CoV-2-Infektion und eines Schlaganfalls bedingte Mortalität beträgt mehr als 15–30 %. Während es in manchen europäischen Regionen zu erheblichen Einschränkungen der Akutversorgung von Schlaganfällen gekommen ist, sind die Behandlungsraten für die Rekanalisationstherapie in Deutschland während der 1. Pandemiewelle weitgehend stabil geblieben. Es stellten sich aber 20–30 % weniger Patienten mit v. a. leichteren Schlaganfällen und transitorischen ischämischen Attacken in Krankenhäusern vor. Die vorliegende Übersichtsarbeit fasst die aktuellen Erkenntnisse zur Epidemiologie und Pathogenese COVID‑19-assoziierter Schlaganfälle zusammen und beschreibt die bisherigen Auswirkungen der Pandemie auf die Schlaganfallakutversorgung.

## Abstract (en)

Even early at the beginning of the coronavirus disease 2019 (COVID‑19) pandemic, stroke was described as a manifestation or complication of infections with severe acute respiratory syndrome coronavirus 2 (SARS-CoV-2). Current meta-analyses reported a stroke rate of approximately 1.5%. Stroke in COVID‑19 positive patients occurs more frequently in severe courses of the infection and in older patients with cardiovascular comorbidities; however, young patients without cardiovascular risk factors are also not uncommonly affected. The mechanisms of stroke are predominantly embolic. The thrombi frequently occlude large intracranial vessels and in more than 20% affect multiple vascular territories, whereas infarctions due to small vessel disease are uncommon. The exact source of the embolism remains cryptogenic in more than 40% of patients. The mortality caused by the co-occurrence of a SARS-CoV‑2 infection and a stroke exceeds 15–30%. While acute stroke treatment was severely affected in some European regions, the rates of recanalization treatment in Germany largely remained stable during the first pandemic wave; however, 20–30% fewer patients with minor stroke and transient ischemic attacks (TIA) presented to hospitals during the first wave in spring 2020. The present narrative review summarizes the current evidence regarding the epidemiology and pathogenesis of stroke associated with COVID‑19 and describes the effect of the pandemic so far on the provision of acute stroke treatment.

## Hintergrund

Bereits wenige Monate nach dem Ausbruch der durch das „severe acute respiratory syndrome corona virus type 2“ (SARS-CoV-2) bedingten COVID‑19(„coronavirus disease 2019“)-Pandemie wurde in einer ersten chinesischen Fallserie eine erhöhte Schlaganfallrate bei COVID‑19-positiven Patienten beschrieben [17]. Von 219 COVID‑19-positiven Patienten hatten 11 Patienten (5 %) einen Schlaganfall erlitten, davon 10 einen ischämischen. Große Aufmerksamkeit erlangte bald darauf ein Bericht über 5 junge COVID‑19-Patienten aus New York, die schwere ischämische Schlaganfälle ohne erkennbare Risikokonstellation hatten [23]. Auch aus anderen Ländern wurde über eine Häufung arterieller und vor allem venöser thrombembolischer Komplikationen in Zusammenhang mit SARS-CoV‑2 berichtet. Diese Berichte legten nahe, dass eine Infektion mit SARS-CoV‑2 ähnlich oder sogar häufiger als andere Virusinfektionen Schlaganfälle verursachen oder zumindest auslösen kann. Die Mechanismen der Schlaganfallauslösung in Zusammenhang mit COVID‑19 sind bislang aber nur teilweise verstanden.

In einigen europäischen und amerikanischen Regionen und Metropolen hat die Überlastung der medizinischen Versorgungssysteme durch die COVID‑19-Pandemie zeitweise die etablierten Versorgungswege des Schlaganfalls erheblich beeinträchtigt. Diese Einschränkungen betreffen alle Glieder der Schlaganfallversorgungskette einschließlich der Rettungsdienste, der Notfallambulanzen, der neuroradiologischen Interventionen und der Stroke-Units. Neben der Belastung des Gesundheitssystems haben insbesondere auch Ängste von Patienten vor einer SARS-CoV-2-Infektion im Krankenhaus zu einer verminderten Präsentation von Patienten mit leichten Schlaganfällen und transitorischen ischämischen Attacken (TIAs) geführt.

Die vorliegende Übersichtsarbeit fasst zunächst die bisherigen epidemiologischen Daten zur Häufigkeit von Schlaganfällen und verschiedenen Schlaganfallsubtypen bei COVID‑19 zusammen. Im zweiten Teil werden aktuelle Konzepte zur Schlaganfallpathogenese bei COVID‑19 vorgestellt und ein Vergleich zur Influenza gezogen. Anschließend werden die bisherigen Auswirkungen der COVID‑19-Pandemie auf die akute Schlaganfallversorgung beschrieben. Die sich hieraus ergebenden Konsequenzen werden abschließend erörtert.

## Schlaganfallraten bei COVID‑19-infizierten Patienten

Seit den erwähnten ersten Fallserien sind mehrere systematische Reviews und Metaanalysen zur Häufigkeit eines Schlaganfalls bei COVID‑19-Patienten, zu den Risikofaktoren und zur Prognose erschienen.

Nannoni und Kollegen berücksichtigen in ihrem systematischen Review alle bis September 2020 publizierten Studien, die mindestens 5 COVID‑19-positive Schlaganfallpatienten umfassten. In der Metaanalyse mit insgesamt 108.571 COVID‑19-positiven Patienten aus Asien, Europa und Nordamerika lag die gepoolte Inzidenz eines zerebrovaskulären Ereignisses bei 1,4 % (95 %-Konfidenzintervall[KI] 0,4–8,1 %; [22]). 87,4 % der COVID‑19-positiven Patienten erlitten einen ischämischen Schlaganfall und 11,6 % eine intrazerebrale Blutung. COVID‑19-positive Schlaganfallpatienten waren im Vergleich zu nichtinfizierten Schlaganfallpatienten im Median 6 Jahre jünger und bei Aufnahme schwerer betroffen mit einem im Median um 5 Punkte höheren Punktwert auf der Schlaganfallskala der National Institutes of Health. COVID‑19-Patienten hatten zudem eine signifikant höhere Rate an großen Gefäßverschlüssen (Odds Ratio 2,73) und eine signifikant höhere Mortalität (33,3 vs. 11,6 %; OR 5,21). Bei 52,5 % der Betroffenen lagen die Infarkte in mehreren vaskulären Territorien. Die Schlaganfallursache konnte häufig (44,5 %) bei COVID‑19-positiven Schlaganfallpatienten nicht sicher geklärt werden. 19,1 % der ca. 1200 analysierbaren COVID‑19-positiven ischämischen Schlaganfallpatienten erhielten eine systemische Thrombolyse. Die gepoolte Thrombektomierate der COVID‑19-positiven Schlaganfallpatienten war mit 25,9 % sehr hoch, was zu der signifikant höheren Rate an berichteten großen Gefäßverschlüssen passt.

Ein möglicher Selektions- und/oder Publikationsbias muss berücksichtigt werden

In einer im November 2020 publizierten multizentrischen Fall-Kontroll-Studie aus England, die insgesamt 1470 ischämische und hämorrhagische Schlaganfallpatienten umfasste, zeigten sich im Gegensatz zu den Ergebnissen der o. g. Metaanalyse keine signifikanten Unterschiede bezüglich des Alters COVID-positiver und -negativer Schlaganfallpatienten [24], sodass natürlich bei allen gefunden Assoziationen immer auch ein möglicher Selektions- und/oder Publikationsbias berücksichtigt werden muss. Dafür sprechen auch die Ergebnisse einer weiteren systematischen Übersichtsarbeit, die die Schlaganfallinzidenzen aus Studien bis Mai 2020 bei COVID-positiven Patienten untersuchte [7]. Diese lag mit 1,8 % (95 %-KI 0,9–3,7 %) etwas höher als bei Nannoni et al., es zeigte sich aber auch hier eine sehr hohe Heterogenität der eingeschlossenen Studien. Einschränkend muss aber bei dieser Metaanalyse erwähnt werden, dass sie nur 3306 COVID-positive Patienten umfasste. Auch in dieser Metaanalyse war die Mortalität mit 34,4 % (95 %KI 27,2–42,4 %) bei den COVID‑19-positiven Schlaganfallpatienten sehr hoch. In einer multizentrischen retrospektiven Analyse von 8163 COVID‑19-Patienten in den USA entwickelten 1,3 % einen ischämischen Schlaganfall verglichen mit 1,0 % ohne COVID‑19. Die Mortalität im Krankenhaus bei Patienten mit ischämischem Schlaganfall war mit 19,4 % (COVID‑19) niedriger als in vorgenannter Metaanalyse und unterschied sich nicht von Nicht-COVID‑19-Patienten (21,6 %; *p* = 0,66; [26]).

Aufgrund eines sehr wahrscheinlichen Publikations- und Berichtsbias soll hier kurz die Arbeit von Bekelis und Mitarbeitern aus dem stark betroffenen Staat New York erwähnt werden, die in ihrer Analyse von Entlassungsdaten von 24.808 Patienten (davon waren 2513 COVID-positiv) in den Monaten Januar bis April 2020 keine positive Assoziation zwischen einer SARS-CoV-2-Infektion und ischämischem Schlaganfall fanden (OR 0,25; 95 %-KI 0,16–0,40; [3]).

Landesweite Daten aus der 1. Pandemiewelle wurden bisher aus Dänemark und Deutschland publiziert. In dem landesweiten dänischen Register, das alle Krankenhausaufnahmen systematisch erfasst, wurde mithilfe des sog. „Self-controlled-case-series“-Studiendesign die Inzidenz des Auftretens eines ischämischen Schlaganfalls und akuten Herzinfarktes anhand der ICD-10(International Statistical Classification of Diseases and Related Health Problems 10)-Kodierungen bestimmt [21]. Insgesamt hatten sich in dem Beobachtungszeitraum der Studie 5119 Patienten mit COVID‑19 infiziert. Von diesen erlitten 44 einen ischämischen Schlaganfall und 18 einen akuten Herzinfarkt. Das ischämische Schlaganfallrisiko in den ersten 14 Tagen nach laborchemischer Diagnosestellung einer COVID‑19-Infektion war 10-fach höher verglichen zu dem vor der Infektion gelegenen Zeitraum von bis zu 180 Tagen. Dieser Unterschied blieb auch statistisch signifikant, wenn der Beobachtungszeitraum nach der Diagnosestellung auf 31 Tage verlängert wurde. Das Risiko, einen akuten Herzinfarkt in den ersten 14 Tagen nach der Diagnosestellung zu erleiden, war ungefähr 5‑fach erhöht.

In Deutschland hatten von 68.913 Patienten, die im Zeitraum von 16.01.2020 bis 15.05.2020 mit der Hauptdiagnose eines ischämischen Schlaganfalls im Krankenhaus aufgenommen wurden, 213 Patienten eine zusätzliche COVID‑19-Infektion als Nebendiagnose [27]. Das Alter der Patienten lag mit 76 ± 20 Jahren nichtsignifikant höher als bei Schlaganfallpatienten ohne COVID‑19-Infektion (74 ± 19 Jahre). Bezüglich der Geschlechterverteilung gab es keine signifikanten Unterschiede zwischen infizierten und nichtinfizierten Schlaganfallpatienten. Ebenso unterschied sich die Häufigkeit der verabreichten intravenösen Thrombolyse bei COVID‑19-positiven Schlaganfallpatienten nicht von der Rate bei nichtinfizierten Schlaganfallpatienten (16,4 vs. 16,2 %), während die mechanische Thrombektomie signifikant seltener bei COVID‑19-Patienten durchgeführt wurde (3,8 vs. 7,7 %). Die Krankenhaussterblichkeit war in Deutschland bei den COVID‑19-positiven Schlaganfallpatienten erheblich erhöht (22,5 vs. 7,7 %). Bei 172 Patienten, die ursprünglich aufgrund einer COVID‑19-Infektion stationär aufgenommen worden waren, wurde die Nebendiagnose eines ischämischen Schlaganfalls kodiert. Die Krankenhaussterblichkeit bei diesen Patienten lag bei 49,4 %. Unter Berücksichtigung beider Konstellationen betrug die Krankenhaussterblichkeit bei COVID‑19-infizierten Patienten mit einem ischämischen Schlaganfall als Haupt- oder Nebendiagnose in Deutschland insgesamt 34,6 %.

Die Mortalität war in der BRD bei COVID‑19-positiven Schlaganfallpatienten erheblich erhöht

Zusammenfassend geht eine SARS-CoV-2-Infektion nach den bisher vorliegenden Daten mit einem leicht erhöhten Schlaganfallrisiko einher. Es treten überwiegend embolische Schlaganfälle in größeren Hirngefäßen auf, die mit einer deutlich erhöhten Mortalität einhergehen. In Tab. 1 sind die wichtigsten epidemiologischen Daten zusammengefasst.

**Table 1:** 

Schlaganfallinzidenz bei COVID‑19-Patienten [22, 26]	Asien	63/2348 → 3,1 % (95 %-KI 1,9–5,1 %)
	Europa	83/7795 → 1,2 % (95 %-KI 0,7–1,9 %)
	Nordamerika	960/98.428 → 1,1 % (95 %-KI 0,8–1,4 %; [22])
	USA	103/8163 → 1,3 % [26]
Demographische Faktoren bei COVID-positiven Schlaganfallpatienten	Alter, Jahre	Median 65,3 (Range 61,4–67,6; [22]) Median 65,0 (Range 54,0–76,3; [9]) Mittel 68,8 (Standardabweichung 15,1; [26])
	Geschlecht (männlich)	62,4 % [22] 57 % [9] 44,7 % [26]
Verteilung zerebrovaskulärer Ereignisse bei COVID-positiven Patienten	Ischämisch	87,4 % (Range 80,1–92,3 %; [22]) 78,8 % [9]
	Hämorrhagisch	11,6 % (Range 10,1–13,3 %; [22]) 15,0 % [9]
	Sinus‑/Hirnvenenthrombose	0,5 % (Range 0,1–2.2 %; [22]) 4,4 % [9]
Ätiologie der ischämischen Schlaganfälle bei COVID-positiven Patienten	Verschluss einer/mehrerer großer hirnversorgender Arterien	OR 2,73 (95 %-KI 1,63–4,57) COVID‑19-pos. vs. COVID‑19-neg. [22]
Thrombolyserate bei COVID‑19-positiven ischämischen Schlaganfallpatienten	Metaanalyse Dez. 2019 bis Sep. 2020	19,1 % bei COVID‑19-pos.; OR 1,06 (95 %-KI 0,64–1,76) COVID‑19-pos. vs. COVID‑19 -neg. [22]
	Metaanalyse Jan. bis Mai 2020	12,9 % bei COVID‑19-pos. [9]
	Deutschlandweit Jan.–Mai 20	16,4 % bei COVID‑19-pos. vs. 16,2 % bei COVID‑19-neg. [28]
	USA Dez. 2019 bis Apr. 2020	1,0 % bei COVID‑19-pos., 1,0 % COVID‑19-neg. [26]
Thrombektomierate bei COVID-positiven ischämischen Schlaganfallpatienten	Metaanalyse Dez. 2019 bis Sep. 2020	25,9 % bei COVID‑19-pos.; OR 1,13 (95 %-KI 0,71–1,80) COVID‑19-pos. vs. COVID‑19-neg. [22]
	Metaanalyse Jan. bis Mai 20	12,1 % bei COVID‑19-pos [9]
	Deutschlandweit Jan. bis Mai 2020	3,8 % bei COVID‑19-pos. vs. 7,7 % COVID‑19-neg. [28]
	USA Dez. 2019 bis Apr. 2020	1,0 % bei COVID‑19-pos., 1,0 % COVID‑19-neg. [26]
Krankenhausmortalität von Patienten mit einer COVID-Infektion und Schlaganfall	Metaanalyse Dez. 2019 bis Sep. 2020	31,5 % (95 %-KI 27,3–36,0 %), OR 5,21 (95 %-KI 3,43–7,90) COVID‑19-pos. vs. COVID‑19-neg. [22]
	Metaanalyse Jan. bis Mai 2020	34,4 % [9]
	Deutschlandweit Jan. bis Mai 2020	34,6 % [24]
	USA Dez. 2019 bis Apr. 2020	19,4 % COVID‑19-pos. vs. 21,6 % COVID‑19 neg. [26]

*COVID‑19 *„coronavirus disease 2019“, *KI *Konfidenzintervall, *neg. *negativ, *pos. *positive

## Mechanismen der Schlaganfallpathogenese bei COVID‑19

Hinweise auf die Pathogenese von Schlaganfällen bei mit SARS-CoV‑2 infizierten Patienten ergeben sich aus epidemiologischen Daten, aus bildgebenden und laborchemischen Befunden und aus dem wachsenden Verständnis der molekularen Pathomechanismen von COVID‑19.

Die Genese von Schlaganfällen ist heterogen und multifaktoriell und hängt von der individuellen Konstellation bei COVID‑19-Patienten ab. Einerseits kann die SARS-CoV-2-Infektion bei Patienten mit vorbestehender kardiovaskulärer Erkrankung ein akutes Schlaganfallereignis auslösen oder begünstigen [31]. Andererseits gibt es Belege dafür, dass die SARS-CoV-2-Infektion über inflammatorische, vaskuläre oder thrombotische Prozesse zu zerebralen Thrombembolien bei zuvor kardiovaskulär gesunden Menschen führen kann.

Das Zusammenspiel inflammatorischer Prozesse begünstigt die Gerinnselbildung

Das SARS-CoV-2-Virus kann direkt oder über inflammatorische Prozesse eine prothrombotische Diathese hervorrufen [10, 12]. Die Aktivierung der Gerinnungskaskade bei Patienten mit schwerer COVID‑19-Erkrankung ist durch den Anstieg von D‑Dimer und Fibrinogen im Plasma belegt. Durch die Aktivierung des Immunsystems kann es zur Freisetzung proinflammatorischer Zytokine [11], einer daraus resultierenden Plaqueruptur und einer Thrombusauflagerung bei Patienten mit einer Arteriosklerose kommen [18]. Auch Antiphospholipidantikörper und eine damit verbundene Hyperkoagulabilität sind bei COVID‑19-positiven Patienten mit multiplen embolischen Infarkten berichtet worden [38].

Das SARS-CoV-2-Virus nutzt den Angiotensin-converting-enzyme-2(ACE2)-Rezeptor, um in die Wirtszellen einzudringen [39]. Der ACE2-Rezeptor wird neben der Lunge auch im Herzen und in Endothelzellen exprimiert. Das Virus kann in Endothelzellen zu einer Dysfunktion und einer prothrombotischen Veränderung der Gefäßinnenschicht führen [36]. Das Herz, eine häufige Quelle embolischer Schlaganfälle auch unter normalen Umständen, gilt ebenfalls als wichtiger Angriffspunkt für das SARS-CoV-2-Virus [7]. Zu den verschiedenen kardialen Manifestationen von COVID‑19 zählen eine Myokarditis, eine inflammatorische oder Stressmyokardiopathie, eine koronare Ischämie und Herzrhythmusstörungen. Ähnlich wie bei den oben genannten Gefäßerkrankungen kann das Zusammenspiel inflammatorischer Prozessen die Gerinnselbildung auch im Herzen begünstigen. Schließlich ist die häufig schwere Hypoxämie bei COVID-assoziierter Pneumonie besonders bedrohlich für Patienten mit hochgradigen, hämodynamisch relevanten extra- und intrakraniellen Stenosen, da ihre vasomotorische Reservekapazität zur Kompensation einer systemischen Hypoxie bereits erschöpft ist.

Ein erhöhtes Schlaganfallrisiko infolge einer viralen Infektion ist kein für COVID‑19 spezifisches Phänomen [31]. Aufschlussreich ist insbesondere der Vergleich mit der Influenza, einer anderen epidemisch auftretenden viralen Atemwegserkrankung. Eine retrospektive Analyse administrativer Daten von akuten Schlaganfällen zwischen 1994 und 2001, einschließlich Subarachnoidalblutungen und TIAs, berichtete einen schwachen Zusammenhang zwischen Influenzaraten und der Schlaganfallinzidenz [8]. Eine Verdreifachung der Influenzarate führte zu einem 6%igen Anstieg der Schlaganfallinzidenz. Eine weitere Studie berichtete ein erhöhtes individuelles Schlaganfallrisiko nach bestätigter Influenzainfektion verglichen mit anderen Infektionen [37]. Allerdings war das Zeitfenster nach der Infektion mit 1 bis 3 Tagen nach dem Influenzanachweis sehr kurz und die Konfidenzintervalle waren sehr groß. Eine Analyse von Registerdaten aus Südlondon, die die Inzidenz in Bezug auf nichtindividualisierte nationale Influenzaüberwachungsdaten auswertete, fand ebenfalls ein kurzes Intervall. Die stärkste Assoziation zwischen Schlaganfall und Influenzainzidenz lag innerhalb einer Woche nach Influenzainfektionen, während nach einem 5‑Wochen-Zeitraum keine Schlaganfallhäufung mehr nachweisbar war [33]. Durch Analyse von Routinedaten aus dem Gesundheitswesen konnte die größte Wahrscheinlichkeit für einen späteren Schlaganfall innerhalb von 15 Tagen nach einer Influenzainfektion ermittelt werden, während nach 60 Tagen kein erhöhtes Risiko mehr vorzuliegen scheint [5].

Das Schlaganfallrisiko ist bei COVID‑19-Infektion im Vergleich zu Influenza erhöht

Vergleichende Analysen von COVID‑19 und Influenza sind derzeit noch rar. Eine Arbeit aus New York, die an zwei Klinikstandorten die Schlaganfallrate bei COVID‑19-Patienten aus der 1. Welle (März bis Mai 2020) mit denen früherer Influenzapandemien (2016 bis Mai 2018) verglich, fand eine deutlich höhere Wahrscheinlichkeit eines ischämischen Schlaganfalls bei Patienten mit COVID‑19-Infektion (31/1916; 1,6 %) verglichen mit Influenza (3/1486, 0,2 %). Nach Adjustierung für generelle Schlaganfallrisikofaktoren war das relative Risiko 7,6-mal höher bei COVID‑19 [19]. In nur rund einem Viertel der Schlaganfallfälle bei COVID‑19 war der akute Schlaganfall der ursprüngliche Grund für die Klinikvorstellung gewesen. Vielmehr traten bei 74 % die Schlaganfallsymptome erst während des Klinikaufenthaltes auf. Die Schlaganfallrate unterschied sich nicht zwischen Patienten mit Symptomen einer Atemwegserkrankung bei Klinikvorstellung und asymptomatischen Patienten mit Identifikation der Infektion im Routinescreening (1,5 vs. 1,9 %). Zwischen den ersten Symptomen der COVID‑19-Infektion und der Schlaganfalldiagnose vergingen im Median 16 Tage (Interquartilsbereich, 5–28; [19]). Die längerfristigen Auswirkungen einer COVID‑19-Infektion auf das Schlaganfallrisiko sind noch nicht bekannt.

Interessant ist, inwiefern die rasch entwickelten Impfstoffe gegen die SARS-CoV-2-Infektion [25] einen Effekt auf die zukünftige Inzidenz kardiovaskulärer Erkrankungen einschließlich des Schlaganfalls haben werden. Bei der Influenza zeigte eine Metaanalyse von Beobachtungsstudien aus den Jahren 2002 bis 2014 ein verringertes Risiko eines Schlaganfalls bei Patienten, die eine Impfung erhalten hatten [16]. Vergleichbare Daten zu neueren Influenzaserotypen und -impfungen fehlen noch. Eine gepoolte Analyse prospektiver Studien berichtete von keiner Reduktion durch eine Influenzaimpfung eines gemeinsamen Endpunkts aus nichttödlichem Myokardinfarkt, nichttödlichem Schlaganfall oder Tod durch vaskuläre Ursache innerhalb eines 2‑Jahres-Beobachtungszeitraum nach einem kürzlich atherosklerotischen Schlaganfallereignis [15]. Eine Metaanalyse von 6 randomisierten klinischen Studien berichtete ein niedrigeres relatives Risiko eines schweren kardiovaskulären Ereignisses nach einem Jahr nach Influenzaimpfung von 0,64 [35].

## Hirnblutungen und Sinusthrombosen

Intrazerebrale und andere intrakranielle Blutungen sind bei COVID‑19-Patienten in Fallberichten und Fallserien berichtet worden. In einer systematischen Übersichtsarbeit waren 40 % der intrazerebralen Hämatome lobär gelegen, 20 % der Patienten hatten bilaterale Blutungen. Allerdings hat nur ein kleiner Anteil der mit einer COVID‑19-Infektion hospitalisierten Patienten eine Hirnblutung [22]. Ein Anstieg der Inzidenz von Hirnblutungen durch eine COVID‑19-Infektion wurde bislang nicht gezeigt [34]. Potenzielle Faktoren, die eine Hirnblutung bei COVID‑19 begünstigen könnten, stellen eine COVID‑19-assozierte Koagulopathie, Organversagen und der Einsatz von Antikoagulanzien dar. Die häufig atypische, bilaterale Lokalisation der Blutungen könnte auf eine COVID‑19-assoziierte Vasopathie hindeuten. Auch sind zerebrale Mikroblutungen bei kritisch kranken COVID‑19-Patienten im Corpus callosum und in subkortikalen Arealen berichtet worden.

Venöse thrombembolische Ereignisse stellen häufige Komplikationen einer COVID‑19-Erkrankung dar. Interessanterweise gibt es auch Fallberichte und Übersichtsarbeiten von COVID‑19-Patienten mit Sinusthrombose oder Thrombose der äußeren und inneren Hirnvenen [2]. Bei einer Hirnblutung sollte daher differenzialdiagnostisch an eine venöse Stauungsblutung gedacht werden. Bei COVID‑19-Patienten mit Kopfschmerzen, epileptischen Anfällen oder einer Vigilanzminderung sollte auch eine venöse Thrombose in Betracht gezogen werden.

## Einfluss der COVID‑19-Pandemie auf die Behandlung des akuten Schlaganfalls

Schon in den ersten Wochen nach den Mitte März 2020 in Deutschland eingeführten Shutdown-Maßnahmen wurde von vielen Kollegen der deutschen Stroke-Units über einen deutlichen Rückgang der stationär behandelten Schlaganfallpatienten berichtet. Inzwischen liegen aus allen fünf dauerhaft bewohnten Kontinenten Daten zu dem Phänomen der „verschwundenen Schlaganfallpatienten“ vor. Eine Metaanalyse, die neun regionale Studien aus China, Europa und den USA mit insgesamt 59.233 Patienten umfasste, ergab, dass es während der COVID‑19-Pandemie zu nur 64 % der vor der Pandemie auftretenden Schlaganfallnotfallalarmierungen und Krankenhauseinweisungen kam [13]. Die Zahl der Thrombolysetherapien verringerte sich um 31 %, die Zahl der Thrombektomien um 22 % im Vergleich zu der Zeit vor der Pandemie.

In Dänemark wurden in den ersten 3 Wochen nach dem Lockdown im März 2020 signifikant weniger Patienten mit einer TIA und einem ischämischen Schlaganfall stationär aufgenommen als in den Jahren 2017 bis 2019. Hingegen zeigte sich kein signifikanter Rückgang bei den im Krankenhaus behandelten Patienten mit einem hämorrhagischen Schlaganfall [6]. Die Krankenhausmortalität bei den aufgenommenen Schlaganfallpatienten unterschied sich nicht signifikant zwischen der Lockdownperiode und den Vergleichszeiträumen.

Patienten mit leichtem Schlaganfall oder TIA stellten sich weniger häufig im Krankenhaus vor

Aus Deutschland gab es die ersten Berichte anhand von Versicherungsdaten der Barmer Versicherung und aus dem bayrischen TEMPiS-Teleschlaganfall-Netzwerk. In der Analyse der Versicherungsdaten aus dem Zeitraum 01.01. bis 31.05.2020 zeigten sich verminderte Aufnahmeraten von TIA- und Schlaganfallpatienten und eine erhöhte Krankenhaussterblichkeit bei Schlaganfallpatienten (9,8 vs. 8,5 %) gegenüber dem gleichen Zeitraum im Jahr 2019 [30]. Im TEMPiS Netzwerk gingen die Empfehlungen zum Einsatz einer intravenösen Thrombolyse bei akuten ischämischen Schlaganfallpatienten nach dem Lockdown Mitte März 2020 im Vergleich zu den 3 vorherigen Jahren zurück [29]. Die Rate der mechanischen Thrombektomien fiel nach dem Lockdown (7,6 %) auf das Niveau der Jahre 2017 bis 2019. Mittels einer Analyse der deutschlandweiten DESTATIS-Abrechnungsdaten konnten Richter und Kollegen zeigen, dass in den 2 Monaten des ersten COVID‑19-bedingten Lockdowns in Deutschland die stationären Aufnahmen von TIA-Patienten im Vergleich zu den beiden Vormonaten um 23 %, die von ischämischen Schlaganfällen um 17,4 % zurückgingen [25]. Dementsprechend kam es zu einer Abnahme der Absolutzahlen der rekanalisierenden Akuttherapien bei ischämischen Schlaganfallpatienten. Die relative Thrombolyserate blieb während des Lockdowns mit 16,5 % aber unverändert, der Anteil der mechanisch thrombektomierten Patienten nahm deutschlandweit im Gegensatz zu den regionalen Zahlen aus TEMPiS sogar leicht während des Lockdowns zu (8,1 vs. 7,7 %). Dies ist wohl damit zu begründen, dass schwerer betroffene Schlaganfallpatienten mit einem großen Gefäßverschluss weiterhin ins Krankenhaus kamen, während sich Patienten mit einem leichteren („Minor“-)Schlaganfall oder einer TIA aus Angst vor einer SARS-CoV-2-Ansteckung weniger häufig im Krankenhaus vorstellten. Ebenso zeigen Triagedaten einer großen neurologischen Notaufnahme eine Abnahme in den nichtdringlichen Triagestufen, bei gleichzeitig stabiler Anzahl an Patienten der dringlichsten Triagestufe [20]. Dafür spricht auch die während der 2‑monatigen 1. Welle um 1 % höhere Krankenhausmortalität ischämischer Schlaganfallpatienten im Vergleich zu Januar/Februar 2020 und dem entsprechenden Zeitraum im Jahr 2019.

Nicht alle Schlaganfallpatienten erhielten in ihren Zentren die sonst übliche Versorgung

Die stabilen Lyse- und Thrombektomieraten in Deutschland während der COVID-Pandemie im Frühjahr sind wahrscheinlich Folge der im Vergleich zu anderen europäischen Ländern deutlich milder verlaufenden ersten Krankheitswelle und der weniger beeinträchtigten Notfall- und Schlaganfallversorgung. Sie sind auch der hohen Anzahl an Stroke-Unit- und Intensivbetten in deutschen Krankenhäusern zu verdanken [4]. In Frankreich und Spanien zeigte sich in schwer betroffenen Regionen eine deutliche Zunahme der „Door-to-needle“-Zeit bei der intravenösen Thrombolyse bzw. der „Bild-zu-Leistenpunktionszeit“ bei der mechanischen Thrombektomie [14, 32]. In einer Umfrage der European Stroke Organisation, auf die 426 Schlaganfallexperten aus 55 Ländern antworteten, wurden von 77 % der teilnehmenden europäischen Kollegen berichtet, dass nicht alle Schlaganfallpatienten die sonst übliche Versorgung in ihren Zentren erhalten konnten [1]. Dies betraf sowohl die Schlaganfallakutversorgung als auch die Rehabilitation.

## Schlussfolgerungen

Angesichts der Neuheit der Erkrankung und der methodischen Limitationen der bisherigen Studien sollten die dargestellten Erkenntnisse zur Assoziation von COVID‑19 und Schlaganfällen als vorläufig betrachtet werden. Dennoch zeichnet sich ab, dass Schlaganfälle ebenso wie andere neurologische Komplikationen zum Spektrum der Manifestationen der COVID‑19-Erkrankung gehören. Da bei COVID‑19 häufig erst im Verlauf Schlaganfälle auftreten, sind ein klinisch-neurologisches Monitoring und die niedrigschwellige Veranlassung bildgebender Diagnostik bei Infizierten angezeigt. Eine generelle Primärprophylaxe von Schlaganfällen bei mit SARS-CoV-2-Infizierten – in Analogie zur Prävention venöser Thrombembolien – zeichnet sich derzeit noch nicht ab. Bei akuten ischämischen Schlaganfällen sollten die etablierten rekanalisierenden Therapien unter Berücksichtigung hygienischer Schutzmaßnahmen erfolgen und die weitere Betreuung gegebenenfalls auf monitorierten Isolierstationen unter Einbeziehung des Schlaganfallteams fortgeführt werden. Die Sekundärprävention nach einem ischämischen Schlaganfall folgt bislang den üblichen Vorgaben, wobei die ätiologische Diagnostik ungewöhnliche Mechanismen der Schlaganfallpathogenese und insbesondere auch eine Analyse von Thrombophilieparametern einbeziehen sollte. Aktuelle Empfehlungen für die Diagnose und Behandlung gibt die Deutsche Gesellschaft für Neurologie gemeinsam mit anderen Fachgesellschaften in der S1-Leitlinie „Neurologische Manifestationen bei COVID‑19.“ Die Effekte der Pandemie auf die längerfristige Sekundärprävention nach einem Schlaganfall sind naheliegend, aber bislang kaum untersucht.

Bei aller angemessenen Aufmerksamkeit für die COVID‑19-Pandemie darf die Behandlung nichtinfektiöser Entitäten wie Herz-Kreislauf‑, Schlaganfall- und Krebserkrankungen nicht vernachlässigt werden. Die erheblichen „Kollateralschäden“ für die klinische Schlaganfallforschung während der Pandemie, die Folge der Streichung oder Verlagerung personeller, ökonomischer und administrativer Ressourcen sowie der Kontaktbeschränkungen in 2020 war, dürfen sich in Zukunft nicht fortsetzen.

## Fazit für die Praxis

- Mit SARS-CoV‑2 („severe acute respiratory syndrome coronavirus 2“) infizierte Patienten scheinen ein gering erhöhtes Schlaganfallrisiko zu haben.
- Die Mehrzahl COVID‑19(„coronavirus disease 2019“)-assoziierter ischämischer Schlaganfälle ist embolisch, verschließt große intrakranielle Gefäße, betrifft multiple Gefäßterritorien und verläuft schwerer. Die Emboliequelle bleibt bei mehr als 40 % unerkannt.
- Obwohl bei SARS-CoV-2-Infizierten auch Hirnblutungen beschrieben wurden, ist unklar, ob es sich um eine Koinzidenz oder um einen pathogenetischen Zusammenhang handelt.
- COVID‑19-positive Schlaganfallpatienten waren in Metaanalysen internationaler Studien signifikant jünger als nichtinfizierte Patienten.
- Die Krankenhausmortalität war bei COVID‑19-positiven Schlaganfallpatienten in einigen Arbeiten gegenüber nichtinfizierten Patienten deutlich erhöht und liegt weltweit bei ca. einem Drittel.
- COVID‑19-Patienten sollten hinsichtlich der Entwicklung von Schlaganfällen überwacht werden. Die Diagnostik sollte auch ungewöhnliche Ursachen in Betracht ziehen.
